# Effect of sacubitril/valsartan on hospital readmissions in heart failure with reduced ejection fraction in Saudi Arabia: A multicenter retrospective cohort study

**DOI:** 10.1097/MD.0000000000038960

**Published:** 2024-07-26

**Authors:** Samiah Alsohimi, Alaa Almagthali, Dena Mandar, Fatmah Ghandourah, Hala AlButi, Samah Alshehri, Ahmed Aljabri, Mohannad Alshibani

**Affiliations:** a King Abdulaziz University Hospital, Jeddah Saudi Arabia; b King Fahad Armed Forces Hospital, Jeddah, Saudi Arabia; c Faculty of Pharmacy, King Abdulaziz University, Jeddah, Saudi Arabia; d Department of Pharmacy Practice, Faculty of Pharmacy, King Abdulaziz University, Jeddah, Saudi Arabia; e Department of Pharmacy Practice, Faculty of Pharmacy, University of Tabuk, Tabuk, Saudi Arabia.

**Keywords:** ACEI/ARB, heart failure, HFrEF, hospitalization, readmission, sacubitril/valsartan, Saudi Arabia

## Abstract

Sacubitril/valsartan is an angiotensin receptor neprilysin inhibitor (ARNI) that has been shown in multiple clinical trials to have clinical benefits and is recommended by major clinical management guidelines as a first-line treatment for heart failure with reduced ejection fraction (HFrEF). The most significant benefit that was observed in clinical trials is its effect in reducing hospital readmissions. However, little evidence supports its effectiveness in practice, especially in Saudi Arabia. A multicenter retrospective cohort study was conducted using the patient medical records at 2 tertiary hospitals in Saudi Arabia. Eligible patients were adults (≥18 years old) with a confirmed diagnosis of HFrEF who were discharged on either sacubitril/valsartan or angiotensin-converting enzyme inhibitors (ACEI)/angiotensin receptor blockers (ARB) in addition to the other recommended therapy for HFrEF. The primary endpoint was the all-cause 30-day readmission rate. The secondary endpoints included all-cause readmissions at 60-day, 90-day, and 12 months. Additionally, 30-day, 60-day, and 90-day readmissions due to HF were evaluated. A total of 398 patients were included in our analysis; 199 (50.0%) received sacubitril/valsartan (group 1), and 199 (50.0%) received ACEI/ARB (group 2). Our results showed that all-cause 30-day readmissions in group 1 were significantly lower than in group 2 (7% vs 25.0%, RR 0.28, 95% Cl 0.16–0.49; *P* < .001). Additionally, the secondary outcomes showed significantly fewer 60-day, 90-day, and 12-month all-cause readmissions were identified in group 1 compared to group 2 (11% vs 30.7%, RR 0.36, 95% CI 0.23–0.56; *P* < .001), (11.6%. vs 32.6%, RR 0.35, 95% CI 0.23–0.55; *P* < .001) and (23.6% vs 51.2%, RR 0.46, 95% CI 0.35–0.62; *P* < .001), respectively. Furthermore, HF readmissions at 30-day, 60-day, and 90-day in group 1 were significantly lower than in group 2 (*P* < .05). Sacubitril/valsartan for the treatment of HFrEF is associated with a significantly lower rate of all-cause readmission as well as HF readmissions compared to ACEI/ARB. These benefits extend up to 12 months post-discharge.

## 1. Introduction

Heart failure (HF) is described as a clinical progressive condition where the heart cannot fill with or pump blood due to structural or functional cardiac disorders.^[1–3]^ HF is one of the most common diseases with a high rate of morbidity and mortality that imposes a substantial burden on patients, caregivers, and healthcare systems. More than 400.000 parents in Saudi Arabia currently live with HF, with an estimated 37,935 new cases annually.^[4]^ Around 25% of HF patients are readmitted 1 month after discharge, and approximately half are readmitted within 6 months.^[5,6]^ Preventing avoidable readmissions has the potential to lower treatment costs and improve the quality of life for patients.^[7–9]^

Beta-blockers, angiotensin-converting enzyme inhibitors (ACEI), angiotensin receptor blockers (ARB), angiotensin receptor-neprilysin inhibitors (ARNI), Sodium-glucose co-transporter-2 (SGLT2) inhibitors, and spironolactone are parts of the guideline-directed medical therapy (GDMT) which is the standard therapies in patients with heart failure with reduced ejection fraction (HFrEF).^[10–12]^ Each drug can be maximized to a higher effective and tolerated dose. In addition, these therapies might be used in combination for better outcomes.^[13]^

Sacubitril/valsartan is a novel Angiotensin Receptor-Neprilysin Inhibitor (ARNI) that has been shown in multiple clinical trials for its clinical benefits and is recommended by the guidelines as a first-line treatment for HFrEF.^[13,14]^ The use of ARNI has significantly improved the N-terminal pro-brain natriuretic peptide (NT-proBNP), HbA1c levels, and left ventricular ejection fraction (EF).^[15,16]^ One of the most significant benefits of using ARNI observed in clinical trials is reducing hospital readmissions caused by HF.^[17,18]^ In the PARADIGM-HF trial, ARNI was compared to ACEI to assess the impact on morbidity and mortality worldwide in patients with HFrEF. ARNI showed a benefit in decreasing the relative risk of cardiovascular death and HF hospital readmission by 20%.^[17]^ Focusing attention on the readmission problem can improve the quality and outcomes of care for HF patients and possibly reduce costs.^[19,20]^

It is worth noting that real-world patients often exhibit more symptoms, poor renal function, and receive suboptimal dosing, leading to worse outcomes.^[21]^ Accordingly, studies on real-world populations with HFrEF were needed. There are limited studies that evaluated the effectiveness of ARNI in reducing hospital readmission in this population, and most of these studies were conducted in the United States of America.^[22,23]^ Two studies in Asia reported the rate of hospital readmission after using ARNI in HFrEF patients.^[24,25]^ Moreover, readmissions may be related to a variety of care deficits such as premature hospital discharge, inadequate preparation of the patient and their family for discharge, complications that manifest after discharge, or poor care transitions.^[8]^ However, there are no studies about the population of the Kingdom of Saudi Arabia (KSA). Thus, to address this gap, we conducted this study to estimate and compare the readmission rates among patients with HFrEF who were treated with ARNI compared to ACEI/ARB in Saudi Arabia.

## 2. Methods

### 2.1. Study design

This study was a multicenter retrospective cohort study record review including adult patients with a confirmed diagnosis of HFrEF at 2 tertiary hospitals in the KSA. We collected the data from 01-01-2020 to 30-01-2022. The study adhered to the guidelines outlined in the Standard Reporting System for Observational Studies (STROBE).^[26]^ The STROBE checklist was incorporated into our study reporting to ensure transparency and methodological rigor.

### 2.2. Study participants

Patients were identified via reports from the electronic medical record systems. We included adult patients (≥18 years old) with a confirmed diagnosis of HFrEF and EF ≤ 40% based on a recent Echocardiogram, who were discharged on either sacubitril/valsartan or ACEI/ARB plus other recommended therapy for HFrEF. Exclusion criteria were patients who expired during the study period or those with missing data. Eligible patients were separated into 2 groups; the first group received sacubitril/valsartan plus standard of care (group 1), and the second group received ACEI or ARB plus standard of care (group 2).

### 2.3. Data collection

After receiving proper training, the medical record, as well as the physician progress notes were reviewed to obtain the data. Each patient data was abstracted using a standardized data collection tool. The following data were collected: age, gender, body mass index, baseline serum creatinine, systolic blood pressure, diastolic blood pressure, concomitant medications, NT-proBNP, ejection fraction, Platelet, length of stay, serum potassium level, comorbidities (hypertension, coronary artery disease, diabetes mellitus, atrial fibrillation, stroke, chronic kidney disease, hypothyroidism), and concomitant medications.

### 2.4. Study outcomes

The primary endpoint was the all-cause 30-day readmission rate. The secondary endpoints were the 60-day, 90-day, and 12-month all-cause readmission and the 30-day, 60-day, and 90-day readmission due to HF.

### 2.5. Statistical analysis

To detect a difference of 15% in the 30-day readmission between the 2 groups with 80% statistical power and a significance level of 5%, a total of 398 patients would be required to reach the study power. All data were handled according to best practices for raw data management to detect any inaccuracies by statistical analysis.^[27]^ In order to decrease inaccuracies, all interval variables were checked and interpreted in maximum and minimum values. All data were checked and compared against the nominal maximum and minimum value of each variable, and valuable with implausible values would be highlighted. Continuous variables are represented by using mean ± SD, and categorical variables will be represented as (n) and percentages. The Chi-square test was used to test the significance of categorical variables. A t-test was used for normally distributed continuous variables and the Mann-Whitney test was used for non-normally distributed data median, interquartile range. The significance was set at the priority of < 0.05. Data was filled into an appropriately designed Excel sheet, and the statistical analysis was carried out by using IBM SPSS version 21 (IBM Corp., Armonk, NY).^[28]^

### 2.6. Ethical consideration

Due to the study retrospective observational nature, informed consent from study participants was not required.^[29, 30]^ This project was approved by the institutional review boards at both institutions, Reference No 131-21 and REC 429.

## 3. Results

### 3.1. Description of the study cohort

**Figure 1.**
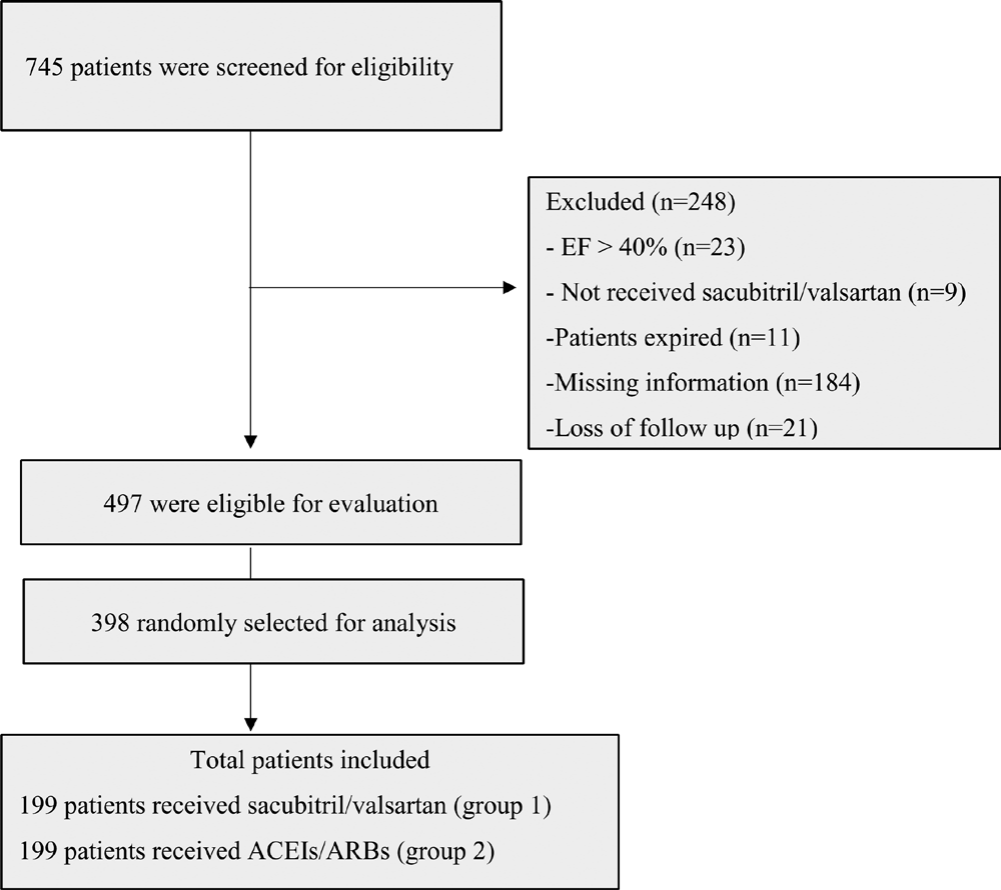
Flowchart of patients’ eligibility screening.

Out of 745 screened patients, 248 were excluded due to mostly missing data (n = 184). Others excluded were EF > 40% (n = 23), loss of follow up (n = 21), patients expired (n = 11), and not received sacubitril/valsartan (n = 9). A total of 398 eligible patients were randomly included in the final analysis (group 1 = Sacubitril/valsartan and group 2 = Other ACEI/ARB) with 199 patients per group (Fig. 1).

The patients’ baseline characteristics are presented in Table 1. The mean age of the 2 groups was comparable (group 1 = 59.9 ± (13.3%) vs in group 2 = 50.5 ± (12.9%); *P* = .60). The most common underlying comorbidities were identified as hypertension 306 (76.8%), followed by diabetes mellitus 282 (70.8%). There was no statistical difference in distribution of comorbidities among the 2 treatment groups (*P* > .05). Overall, all patients received other drugs of the GDMT. A total of 189 (94.9%) vs 185 (92.9%) patients were on B-blocker (*P* = .40), 24 (12%) vs 35 (17.5%) received hydralazine/isosorbide dinitrate (*P* = .50), 139 (69.8%) vs 139 (69.8%) received aldosterone antagonists (*P* = .70), 33 (16.5%) vs 28 (14%) received SGLT2 inhibitors (*P* = .50), and 21 (10.6%) vs 12 (6%) received digoxin (*P* = .09) on group 1 vs group 2, respectively. The most prescribed medications were beta-blockers (94.0%), followed by loop diuretics (93.2%).

**Table 1:** Baseline characteristics of study participants.

Variable	Sacubitril/valsartan (Group 1) (n = 199)	ACEI/ARB (Group 2) (n = 199)	*P* value
Age (yr), mean (± SD)	59.9 (13.3)	50.5 (12.9)	.60
Male, n (%)	148 (74.3)	142 (71.3)	.49
BMI (kg/m^2^), mean (± SD)	29.9 (7.0)	28.9 (5.8)	.14
Baseline serum creatinine (mmol/L), (median, IQR)	81.5 (94–103)	81 (95–128)	.66
Systolic blood pressure (mm Hg), mean (± SD)	127.9 (20.4)	128.7 (21.3)	.58
Diastolic blood pressure (mm Hg), mean (± SD)	74.3 (13.6)	76 (13.1)	.25
NT-proBNP (pg/mL), (median, IQR)	1455 (871–3702)	1835 (1176–2449)	.81
Ejection fraction (%), (median, IQR)	20 (25–30)	20 (25–30)	.05
Platelet (median, IQR)	188.3 (228–272.8)	182.5 (232–301)	.60
LOS (median, IQR)	3 (4–7)	2 (5–9)	.46
Serum K (median, IQR)	3.8 (4–4.5)	3.7 (4–4.6)	.17
Hypertension, n (%)	149 (74.8)	157 (79.2)	.29
myocardial infarction, n (%)	49 (24.6)	36 (18.1)	.12
Diabetes mellitus, n (%)	143 (71.8)	139 (70.2)	.71
Atrial fibrillation, n (%)	36 (18.1)	36 (18.1)	1.00
Stroke, TIA, n (%)	15 (7.5)	15 (7.5)	.98
CKD, n (%)	20 (10)	33 (16.6)	.05
Hypothyroidism, n (%)	15 (7.5)	15 (7.5)	1.00
Thiazide diuretics, n (%)	8 (4)	6 (3)	.58
Loop diuretics, n (%)	186 (93.4)	185 (92.9)	.84
BB, n (%)	189 (94.9)	185 (92.9)	.40
Hydralazine/ISDN, n (%)	24 (12)	35 (17.5)	.48
Aldosterone antagonists, n (%)	136 (68.3)	139 (69.8)	.74
SGLT2 inhibitors, n (%)	33 (16.5)	28 (14)	.48
Digoxin, n (%)	21 (10.6)	12 (6)	.09

ACEI = angiotensin-converting enzyme inhibitor, ARB = angiotensin receptor blocker, BB = beta blocker, IQR = interquartile range, ISDN = isosorbide dinitrate, LOS = Length of stay, n = number, SD = standard deviation, SGLT2 = sodium-glucose cotransporter, TIA = transient ischemic attack, NT-proBNP = N-terminal pro-brain natriuretic peptide.

### 3.2. Sacubitril/valsartan and ACEI/ARB readmission outcomes

#### 3.2.1. All-cause 30-day readmission

The primary outcome of all-cause 30-day readmission was significantly lower in the sacubitril/valsartan compared to the ACEI/ARB group (7% vs 25%, RR 0.28, 95% CI 0.16–0.49; *P* < .001, respectively) (Table 2).

**Table 2:** Effects of sacubitril/valsartan and ACEI/ARB on readmission outcomes.

Study outcomes			Sacubitril/valsartan (Group 1) (n = 199)	ACEI/ARB (Group 2) (n = 199)	Relative risk (RR)	95% Confidence Interval (Cl)	*P* value
Primary outcome	All-cause readmission	30-d, n (%)	14 (7.0)	50 (25.1)	0.28	0.16–0.49	<.001
Secondary outcomes	All-cause readmission	60-d, n (%)	22 (11.0)	61 (30.7)	0.36	0.23–0.56	<.001
		90-d, n (%)	23 (11.6)	65 (32.6)	0.35	0.23–0.55	<.001
		12-mo, n (%)	47 (23.6)	102 (51.2)	0.46	0.35–0.62	<.001
	HF readmission	30-d, n (%)	10 (5.0)	34 (17.0)	0.29	0.15–0.58	<.001
		60-d, n (%)	16 (8.0)	41 (20.6)	0.39	0.23–0.67	<.001
		90-d, n (%)	19 (9.5)	54 (27.1)	0.35	0.22–0.57	<.001

ACEI = angiotensin-converting enzyme inhibitor, ARB = angiotensin receptor blocker.

#### 3.2.2. All-cause 60-day, 90-day, and 12 months readmission

The secondary outcomes of all-cause readmissions 60-day, 90-day, and 12 months on sacubitril/valsartan plus standard of care were significantly lower than ACEI/ARB plus standard of care group (11% vs 30.7%, RR 0.36, 95% CI 0.23–0.56; *P* < .001), (11.6%. vs 32.6%, RR 0.35, 95% Cl 0.23–0.55; *P* < .001) and (23.6% vs 51.2%, RR 0.46, 95% Cl 0.35–0.62; *P* < .001), respectively (Table 2).

#### 3.2.3. Readmission due to HF at 30-day, 60-day, and 90-day

Of the 199 patients who received sacubitril/valsartan, the rate of 30-day, 60-day, and 90-day HF readmission were also significantly lower compared to the ACEI/ARB group (5% vs 17%, RR 0.29, 95% CI 0.15–0.58; *P* < .001), (8% vs 20.6%, RR 0.39, 95% CI 0.23–0.67; *P* < .001) and (9.5% vs 27.1%, RR 0.35, 95% CI 0.22–0.57; *P* < .001), respectively (Table 2).

## 4. Discussion

In this multicenter retrospective study involving adult patients with HFrEF, we found that all-cause 30-day readmissions in real-world practice for sacubitril/valsartan plus standard of care were significantly lower compared to ACEI/ARB plus standard of care. Simultaneously, all-cause readmissions at 60-day, 90-day, and 12 months, as well as the 30-day, 60-day, and 90-day readmissions due to HF in the sacubitril/valsartan group, were significantly lower than ACEI/ARB. To our knowledge, this is the first multicenter study in KSA that investigated the efficacy of sacubitril/valsartan plus standard of care in reducing hospital readmission in patients with HFrEF.

ARNI, sacubitril/valsartan, was addressed by major guidelines as a frontline treatment for HFrEF. PARADIGM-HF trial was a prospective randomized double-blinded multicenter study evaluating the impact of ARNI compared to ACEI in reducing cardiovascular death and hospital readmission in patients with HFrEF.^[17]^ Out of more than 8000 included patients, ARNI significantly reduced the composite endpoint of cardiovascular death and hospital readmission compared to ACEI/ARB over a mean duration of follow-up around 27 months (21.8% vs 26.5%, Hazard ratio [HR] 0.80; 95% CI 0.73–0.87, *P* < .001).^[17]^ ARNI group had a rate of HF readmission of 12.8% compared to 15.6 % in the other group (HR 0.79; 95% CI 0.71–0.89, *P* < .001).^[17]^ It should be noted that all-cause readmissions were not evaluated in the PARADIGH-HF trail. According to the positive results of this clinical trial, the drug was granted the US Food and Drug Administration approval for HFrEF.^[17]^ To validate the results outside the tightly controlled settings, a few studies were conducted to evaluate the impact of ARNI on the rate of hospital readmission in real-world patients where routine clinical practice is provided to the patients. The majority of these studies included US patients,^[22,23]^ with 2 studies reporting the rate of readmission in Taiwan^[24,25]^ and one in Ireland.^[31]^

Most of these studies evaluated the rate of both all-cause and HF readmissions with a mean follow-up period ranging from 1 month up to 14 months. Sacubitril/valsartan was associated with a significantly lower rate of hospital readmissions in most of the studies with a relative risk reduction ranging between 3% and 44%, which were extended up to 14 months post-discharge.^[32–34]^ The magnitude of the reduction was higher in all-cause readmissions compared to HF readmissions. It should be noted that males were predominant in these trials.

The highest relative risk reduction was reported by Albert et al. This study utilized retrospective claims data for patients with HFrEF to evaluate the impact of sacubitril/valsartan on the rate of all-cause readmissions and HF readmissions compared to ACEI/ARB. They included a total of 558 patients with a mean age of 68 years old. Over a mean duration of around 6 months, they found that the rate of all-cause readmissions and HF readmissions were significantly reduced by 43% and 44%, respectively (HR 0.57, 95% Cl 0.42–0.77, *P* < .001; HR 0.56, 95% CI 0.33–0.94, *P* = .03, respectively).^[33]^ On the other hand, Greene et al reported the lowest relative risk reduction of all-cause readmissions in patients discharged on sacubitril/valsartan vs ACEI/ARB.^[23]^ The authors of this study included 9426 Medicare patients aged > 65 years old with HFrEF to evaluate the difference in the rate of hospital readmissions up to 12 months between the 2 medications. The median age in this study was 78 years, which represents the highest median age among other studies. While the adjusted relative risk of all-cause readmission was lower in sacubitril/valsartan (HR 0.97, 95% CI 0.89–1.07, *P* = .55), the adjusted relative risk of HF readmission was higher in this group compared to ACEI/ARB group (HR 1.04, 95% CI 0.91–1.18, *P* = .59).^[23]^ This result is consistent with the subgroup analyses of Tan et al, which found out the rate of HF readmissions was lower in young patients (HR 0.87, 95% CI 0.7–1.08) but not in patients aged ≥ 75 (HR 1.21, 95% CI 1.02–1.44).^[35]^

Consistent with the PARDIGH-HF study and other real-world studies, our study confirms the benefits of ARNI over ACEI/ARB in minimizing the risk of all-cause readmissions as well as HF readmissions, which extended up to 12 months. In fact, our study reported the highest relative risk of reduction in both outcomes as the relative risk reduction of all-cause readmissions 30-day, 60-day, 90-day, and 12 months post discharged were 72%, 64%, 65%, and 54%, respectively. Similarly, the relative risk reduction of HF readmissions 30-day, 60-day, and 90-day were 71%, 61%, and 65%, respectively. These results could be explained by the fact that the mean age of patients in our study was 55 years, which is considered the lowest mean age among other studies. Moreover, the management of HFrEF in our patients followed the GDMT in most of the cases. Carnicelli et al concluded that high adherence to sacubitril/valsartan within 90 days post-discharge is associated with a substantially lower rate of readmissions in patients with HFrEF.^[22]^ While we did not assess adherence to the medications, it is a possibility that the medication adherence was high among our patients, which may contribute to the noticed high reduction in readmission rates. The findings of our study are quite interesting since the reported rate of hospital readmission in patients with a history of HFrEF in KSA can be as high as 37%.^[36]^ Little is known about the effectiveness of sacubitril/valsartan in KSA, and the results of our study could have a meaningful impact on the current practice in Saudi Arabia.

However, our study was not free of limitations. First, a retrospective study might not capture all eligible patients. Second, target medication doses and stage of HF were not evaluated. Finally, the data on hospital readmissions to other hospitals were not available, which could lower the reported readmission rates.

## 5. Conclusion

The utilization of sacubitril/valsartan for the treatment of HFrEF is associated with a significantly lower rate of all-cause and HF readmissions compared to ACEI/ARB. These benefits extend up to 12 months post-discharge. Further studies from different regions in KSA are needed to generalize the results.

## Author contributions

**Conceptualization:** Samiah Alsohimi, Dena Mandar, Fatmah Ghandourah, Hala AlButi, Ahmed Aljabri, Mohannad Alshibani.

**Data curation:** Samiah Alsohimi, Alaa Almagthali, Mohannad Alshibani.

**Formal analysis:** Samah Alshehri, Mohannad Alshibani.

**Investigation:** Dena Mandar, Fatmah Ghandourah, Hala AlButi, Samah Alshehri, Ahmed Aljabri.

**Methodology:** Alaa Almagthali, Dena Mandar, Fatmah Ghandourah, Hala AlButi, Samah Alshehri, Ahmed Aljabri, Mohannad Alshibani.

**Resources:** Samiah Alsohimi.

**Software:** Samah Alshehri.

**Supervision:** Dena Mandar, Fatmah Ghandourah, Hala AlButi, Samah Alshehri, Ahmed Aljabri, Mohannad Alshibani.

**Validation:** Samiah Alsohimi, Alaa Almagthali, Ahmed Aljabri.

**Writing – original draft:** Samiah Alsohimi.

**Writing – review & editing:** Alaa Almagthali, Dena Mandar, Fatmah Ghandourah, Hala AlButi, Samah Alshehri, Ahmed Aljabri, Mohannad Alshibani.
